# Toxicity and antioxidant effects of the decoction of the leaf of guavira (*Campomanesia guazumifolia*) as a food additive for *Geophagus pyrocephalus*

**DOI:** 10.1007/s11259-026-11270-9

**Published:** 2026-05-19

**Authors:** Marcos Paiva Scardua, Maria Ildilene da Silva, Antonio Cesar Godoy, Wesley Barbieri, Frederico Vasconcelos, Taline Baganha Stefanello Catelan, Claudia Andrea Lima Cardoso, Heriberto Gimênes, Dacley Hertes Neu, Claucia Aparecida Honorato

**Affiliations:** 1 Federal University of Grande Dourados (UFGD), Dourados, Mato Grosso do Sul Brazil; 2 Federal University of Mato Grosso do Sul (UFMS), Campo Grande, Brazil; 3 University Center of Grande Dourados (UNIGRAN), Dourados, Brazil; 4 State University of Mato Grosso do Sul (UEMS), Dourados, Brazil

**Keywords:** Welfare, Guavira, Ornamental aquaculture, Health

## Abstract

The use of plant-derived nutraceuticals in aquaculture has increased, including in ornamental species. Guavira (*Campomanesia guazumifolia*) is a native Cerrado plant with reported antioxidant and anti-inflammatory properties. This study evaluated the toxicity and functional effects of *C. guazumifolia* leaf decoction as a feed additive for *Geophagus pyrocephalus*. Acute toxicity assays were conducted using *Artemia salina* (1.5–1000 µg mL⁻¹) and juvenile fish (0.1–500 µg mL⁻¹), alongside a 30-day feeding trial with dietary inclusion levels of 0, 250, 500, 1000, and 2000 mg kg⁻¹. The decoction exhibited high toxicity, with LD₅₀ values of 23.29 µg mL⁻¹ for *A. salina* and 50.03 µg mL⁻¹ for *G. pyrocephalus*. In fish, mortality increased markedly at concentrations ≥ 100 µg mL⁻¹, accompanied by elevated plasma glucose and severe gill lesions, including epithelial hyperplasia, lamellar fusion, and necrosis. Despite this toxicity, dietary supplementation at 250 mg kg⁻¹ significantly enhanced muscle antioxidant activity, with superoxide dismutase (SOD) levels increasing by approximately 2.5-fold compared to the control (*p* < 0.05), without affecting growth performance or survival. In contrast, higher inclusion levels (≥ 1000 mg kg⁻¹) were associated with reduced condition factor and increased hepatic alanine aminotransferase (ALT), indicating metabolic stress. In conclusion, *C. guazumifolia* leaf decoction presents a narrow safety margin, combining pronounced toxicity at relatively low concentrations with beneficial antioxidant effects at low dietary inclusion. The dose of 250 mg kg⁻¹ is suggested as a safe and functional level for *G. pyrocephalus*, highlighting the importance of dose optimization for its application in ornamental aquaculture.

## Introduction

Medicinal plants and their derivatives are rich sources of diverse bioactive constituents with a range of biological activities (Carvalho and Conte-Junior 2021). Plant-derived products have been widely used in traditional medicine as preventive and therapeutic alternatives (Al-Okbi et al. 2014; Barnes 2020; Manzoor et al. 2022). In this context, species from the Brazilian Cerrado have been investigated for their antiplatelet potential and are widely used in food and beverage preparation (Lescano et al. 2018, 2021; Tolouei et al. 2020). The Brazilian Cerrado is a tropical savanna biome located in central Brazil, recognized as one of the world’s biodiversity hotspots, with high levels of endemism and significant ecological importance (Ribeiro and Walter 2008). Particular attention has been given to phenolic compounds such as flavonoids, which are abundant in nature and exert diverse effects on antiplatelet agents (Lamponi 2021; Fernández-Rojas et al. 2022).

The Brazilian Cerrado is a biome characterized by high biodiversity of native species, recognized for its medicinal, nutraceutical, and food potential (Carvalho and Conte-Junior 2021). Among these species, *Campomanesia guazumifolia* (Cambess.) O Berg stands out (commonly known as sete-capotes, sete-capas, capoteira, sete-casacas, arázeiro, araçá-do-mato) and is traditionally used to treat intestinal and hepatic disorders, also showing anti-inflammatory and anti-edematogenic actions (Dorigoni et al. 2001).

Studies have shown that leaf infusion of *C. guazumifolia* exhibits anti-inflammatory activity (Catelan et al. 2018), while the ethanolic extract presents photoprotective potential (Catelan et al. 2019). These biological activities are mainly attributed to phenolic compounds found in the plant (Cialdella-Kam et al. 2017; Catelan et al. 2018), which may be explored as feed additives to improve animal health (Viscardi et al. 2017).

In aquaculture, various plant-derived ingredients have been incorporated into diets to promote fish health (El-Barbary 2018; Li et al. 2022; Moraes et al. 2025). However, despite the growing interest in plant-derived additives in aquaculture, there is a lack of information regarding the safety, dose-dependent toxicity, and physiological effects of *Campomanesia guazumifolia* in fish, particularly ornamental species (Siqueira et al. 2024). Additionally, the potential dual role of its bioactive compounds as antioxidants at low doses and toxic agents at higher concentrations remains poorly understood (Reges et al. 2024).

Ornamental species, especially cichlids, are valued in aquaculture for their aesthetic appeal and market acceptance (Karsli 2021). *Geophagus pyrocephalus* is a Brazilian cichlid known for its intense red cephalic coloration, which is a key commercial trait for aquarists (Luo et al. 2021; McLean 2021; Vissio et al. 2021; Lau et al. 2023; Scardua et al. 2024).

Therefore, this study aimed to investigate the oral toxicity and effects of *C. guazumifolia* leaf decoction when included in the diet of *G. pyrocephalus*, in order to assess its potential as a functional feed additive for ornamental fish.

## Materials and methods

### Biological material

**Fig. 1:**
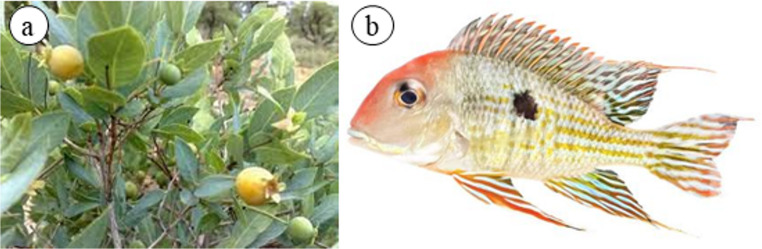
**a** Image of *C. guazumifolia*, personal archive. **b** *G. pyrocephalus*, photo by Guilherme Pereira Marconato

Leaves of *C. guazumifolia* (Fig. 1A) were collected on a farm located in the municipality of Amambai, Mato Grosso do Sul. The species was identified and deposited in the Herbarium of the Federal University of Grande Dourados (UFGD), Dourados-MS, under number 4746. Leaves were dried in a circulating-air oven at 37 ± 2 °C and milled in a Wiley-type mill (Marconi) using a 10-mesh sieve. The sample was then packaged, labeled and stored at room temperature.

The fish, *G. pyrocephalus* (Fig. 1B), were obtained from Bioparque Pantanal, located in Campo Grande, Mato Grosso do Sul, Brazil. The experiment was approved by the Animal Research Ethics Committee, University Center of Grande Dourados (UFGD) under protocol number 003/2014. SISGEN registration code: AA88DF1.

Decoction of *C. guazumifolia* was obtained employing leaves at a rate of 20 g L^− 1^ remained in contact with water 100 °C for 10 min in an enclosed container (Vinagre et al. 2010). The decoction was filtered and subsequently lyophilized at −42 °C under vacuum (0.045 mbar). Extraction yield was calculated based on the dry lyophilized mass, and the extract was stored at −20 °C. Chemical composition was evaluated at a concentration of 1 mg g^− 1^ of extract.

Phenolic compound content was determined using the Folin-Ciocalteu colorimetric method (Djeridane et al. 2006). For this procedure, 0.5 mL of Folin-Ciocalteu reagent (1:10 v: v) and 1000 µL of distilled water were added to 100 µL of each sample and allowed to stand for 1 min. Then 1.5 mL of 20% aqueous sodium carbonate solution was added and the reaction was kept in the dark for 120 min. Absorbance was read at 760 nm. Results were expressed as mg of gallic acid equivalents (GAE) per g of extract.

The leaves infusion of *C. guazumifolia* lyophilized (ICG) was initially subjected to clean up using C-18 SPE cartridges (50.0 mg) (Strata C18-E, from phenomenex) which was activated with 4.0 mL of methanol. The sample (3.0 mg) was solubilized in the least possible amount of mobile phase and subsequently subjected to solid phase extraction, with 5.0 mL of methanol. The obtained solution was dried at room temperature, redissolved in methanol, reaching the concentration of 3.0 mg/mL and filtered in 0.45 μm filter (CHROMAFIL^®^ Xtra). Then, the sample was subjected to analysis. Direct injection was performed (FIA-ESI-IT-MS, negative mode – Mass spectrometer LTQ XL equipped with an APCI ionization source and linear ion-trap analyzer) to obtain the mass spectra in negative mode [M–H]-, collision energy for each fragmentation was 35%, and experiments were carried out for major peaks MS2 and MS3. It was performed a scan of m/z (mass-to-charge ratio) 50–2000; the parameters of the equipment were: capillary temperature of 3000 °C, capillary voltage 13 V, spraying voltage 5 kV, sheath gas flow rate 35 (nitrogen, arbitrary units), and auxiliary gas flow rate 10 (arbitrary units).

### Toxicity assessment

The study was conducted in accordance with biosafety regulations and the Ethical Principles in Animal Experimentation established by the National Council for the Control of Animal Experimentation (CONCEA).

The experimental design followed a completely randomized design. Two independent bioassays were conducted: (i) toxicity test using Artemia salina and (ii) acute toxicity test in juveniles of *Gymnocorymbus pyrocephalus.*

Toxicity test using Artemia salina - The toxicological assay with Artemia salina was performed according to Meyer et al. (1982). Artemia cysts were incubated for 48 h in synthetic seawater (20 g L⁻¹) supplemented with 0.7 g L⁻¹ sodium bicarbonate (pH 8.0), under continuous illumination (60 W) and constant aeration. To determine the median lethal concentration (LC₅₀), organisms were exposed to eight concentrations of the aqueous extract of *C. guazumifolia*: 0.0 (control), 1.5, 5.0, 25.0, 125.0, 250.0, 500.0, and 1000.0 µg mL⁻¹. Each treatment consisted of three replicates (*n* = 3), with 10 individuals per replicate, totaling 30 organisms per concentration. A negative control (saline solution) was included. After 24 h of exposure, mortality was recorded, and the percentage of dead individuals was calculated for each concentration.

Acute toxicity assay in fish - Juveniles of *G. pyrocephalus* (5.53 ± 1.04 g) were used in the acute toxicity test. Fish were acclimated for seven days in 60 L tanks at a stocking density of 10 fish per experimental unit, under constant aeration, temperature of 27 ± 1 °C, and a 12 h light:12 h dark photoperiod (Godoy et al. 2021). During acclimation, fish were fed twice daily to apparent satiation with a commercial extruded diet containing 32% crude protein (Fujiomoto et al. 2016). After acclimation, fish were exposed for 24 h to the following concentrations of the aqueous extract: 0.0 (control), 0.1, 1.0, 10.0, 25.0, 50.0, 100.0, 200.0, 300.0, 400.0, and 500.0 µg mL⁻¹.

The assay consisted of ten replicates per treatment (*n* = 10), with each replicate corresponding to one aquarium (60 L) containing 10 fish, totaling 100 fish per treatment. The mass-to-volume ratio was maintained at approximately 1 g L⁻¹, following ABNT NBR 15088:2011 recommendations (Associação Brasileira de Normas Técnicas 2011). Feeding was suspended 24 h prior to the experiment. Water quality parameters (temperature, dissolved oxygen, turbidity, and pH) were monitored throughout the experimental period (Godoy et al. 2021).

Fish mortality was recorded as an indicator of tolerance to the extract. Observations were performed hourly during the first six hours and subsequently at 6-hour intervals until the end of the 24-hour exposure period. At the end of the exposure, all fish from each experimental unit were sampled. Blood was collected from the caudal vein for glucose determination (Trinder 1969).

After blood collection, five fish were anesthetized with eugenol (100 mg L⁻¹) (Honorato and Nascimento 2016) and gill samples were collected for histopathological analysis. Tissue samples were fixed in 10% buffered formalin for 24 h and then transferred to 70% ethanol. Subsequently, samples were dehydrated in an ascending ethanol series, cleared, and embedded in paraffin (Histosec^®^, Merck). Sections of 2–5 μm thickness were obtained and stained with hematoxylin and eosin (H&E). Microscopic analyses were performed using an Olympus BX41 photomicroscope, and images were documented.

Histopathological evaluation followed the criteria proposed by Bernet et al. (1999), in which lesions are classified according to reaction patterns (circulatory disturbances, regressive changes, progressive changes, and inflammatory responses). When applicable, lesion severity was assessed to support a semi-quantitative interpretation of tissue alterations.

### Feeding trial

The in vivo feeding trial took place at Bioparque Pantanal (Campo Grande, MS, Brazil) in a completely randomized design (CRD) with five treatments (0, 250, 500, 1000, and 2000 mg kg⁻¹ of *C. guazumifolia* decoction) and three replicates each. A total of 150 G. pyrocephalus juveniles (average 2.74 ± 0.82 g; 5.31 ± 0.46 cm) were distributed in 15 aquaria (30 L) with ten fish each (30 fish per treatment). Each unit had its own recirculation system, filtration, and a 12 h photoperiod.

The experimental diets were prepared using a commercial basal diet (Poytara Ltd., Brazil; 1.0 mm pellets) with the following proximate composition: 40.88% crude protein, 9.6% lipids, 10% ash, 3.5% crude fiber, and 4,374.8 kcal kg⁻¹ of gross energy. To incorporate the extract, the basal diet was ground and mixed with the *C. guazumifolia* decoction for 30 min at room temperature. The pellets were thereafter reformed and dried at 50 °C for 24 h in a forced-air oven. This temperature was selected to ensure moisture removal while maintaining the integrity of phytochemical compounds, as drying below 60 °C is recognized to preserve antioxidant properties (Ota et al. 2019). Diets were stored in amber containers at 4 °C to prevent photodegradation.

Fish were fed to apparent satiation twice daily for 30 days. At the end of the trial, zootechnical performance was evaluated based on weight gain, length gain, and condition factor (Lima-Junior et al. 2002). To ensure representative and statistically robust analyses, three fish were randomly sampled from each replicate (*n* = 9 per treatment), providing adequate power for both univariate and multivariate (PCA) analyses. These fish were anesthetized with eugenol (100 mg L⁻¹), euthanized, and their organs were removed and frozen for analyses of hepatic enzymes, muscle and liver antioxidant activity, and total protein. Samples for histopathological evaluation were also collected and processed according to the criteria proposed by Bernet et al. (1999).

### Enzymatic analyses

For hepatic metabolic enzyme analysis, alanine aminotransferase (ALT) and aspartate aminotransferase (AST) samples of liver (100 mg) were homogenized in sodium phosphate buffer (glycerol v/v in 20 mM sodium phosphate and 10 mM Tris buffer, pH 7.0) using a Potter-Elvehjem homogenizer. Samples were centrifuged at 4 °C for three minutes at 600 × g and the supernatant subjected to a second centrifugation for eight minutes at 6000 × g. The resultant supernatant was used for ALT and AST assays. Enzyme activities were determined by a modification of the Reitman and Frankel method (1957). Readings were performed on a semi-automatic spectrophotometer (Bioplus S-200) at wavelengths appropriate for each test.

Superoxide dismutase (SOD) was assayed by the auto-oxidation of pyrogallol, which is inhibited in the presence of SOD (Beutler 1984; modified). Absorbance readings were performed at 420 nm, where 1.0 IU mg^− 1^ inhibits 50% of pyrogallol auto-oxidation. Catalase (CAT) activity was assessed by monitoring the decay of H_2_O_2_ at 230 nm (Beutler 1984). One CAT unit was defined as the amount of enzyme required to decompose 1.0 µmol of H_2_O_2_ min^− 1^, using an extinction coefficient of ε230 = 0.071 mM cm^− 1^. Total protein was quantified using the Bradford reagent against a BSA standard (Kruger 2009) for both liver and muscle samples.

### Statistical analysis

Toxicity assay results were expressed as mortality indices (%) and analyzed using the non-parametric Kruskal-Wallis test (*p* < 0.05), followed by Dunn’s post-hoc test for multiple comparisons, as these data did not meet the assumptions of normality and homogeneity of variances. The median lethal dose (LD50​) was estimated using probit analysis based on mortality data, with 95% confidence intervals calculated to assess the precision of the estimate. For the feeding toxicity test, a completely randomized design (CRD) with five treatments and three replicates was used. Data normality was independently assessed using the Shapiro–Wilk test, while the homogeneity of variances was verified using Levene’s test. When both assumptions were met, one-way analysis of variance (ANOVA) was performed. When significant differences (*p* < 0.05) were detected, treatment means were compared using Tukey’s test.

A multivariate analysis (Principal Component Analysis - PCA) was used to evaluate correlations between quantitative discrete variables (decoction concentration levels) and quantitative continuous variables (enzymatic data). PCA reduces data dimensionality by forming linear combinations that discard components with low variance, highlighting the most influential variables. PCA was performed in R (R Core Team 2022) using FactoMineR (Husson et al. 2024) and factoextra (Kassambara 2020).

## Results

The decoction of *C. guazumifolia* leaves contained 728.70 ± 13.20 mg GAE g⁻¹ dry extract, as determined by the Folin-Ciocalteu method. This value refers to the lyophilized extract (extraction yield basis), not the raw plant material, which explains the relatively high phenolic concentration. The analyses were performed in negative electrospray ionization mode (ESI⁻), and the detected precursor ions correspond to deprotonated molecular ions [M–H]⁻ of flavonoid glycosides. Fragmentation patterns were interpreted based on neutral losses characteristic of sugar moieties, including pentose (−132 Da), hexose (−162 Da), and deoxyhexose (−146 Da), allowing structural assignment of glycosylated derivatives. Five compounds were identified based on their MS/MS fragmentation patterns (Table 1).

**Table 1:** Tentative identification of flavonoids in the decoction of *Campomanesia guazumifolia* leaves by FIA-ESI-IT-MS in negative ion mode

Precursor ion [M-H]⁻ (m/z)	MS/MS fragment íons (m/z)	Tentative identified	Bibliographic references
301	132	Quercetin pentose	Gondoin et al. 2010 Scoparo et al. 2012 Huang et al. 2015
301	146	Quercetin deoxyhexoside	Simirgiotis et al. 2013 Chavez et al. 2016
316	146	Myricetin deoxyhexoside	Simirgiotis et al. 2013 Chavez et al. 2016
316	163	Myricetin hexoside	Kim et al. 2011 Singh et al. 2011 Fang et al. 2007
316	152	Myricetin deoxyhexoside gallate	Fang et al. 2007

**Fig. 2:**
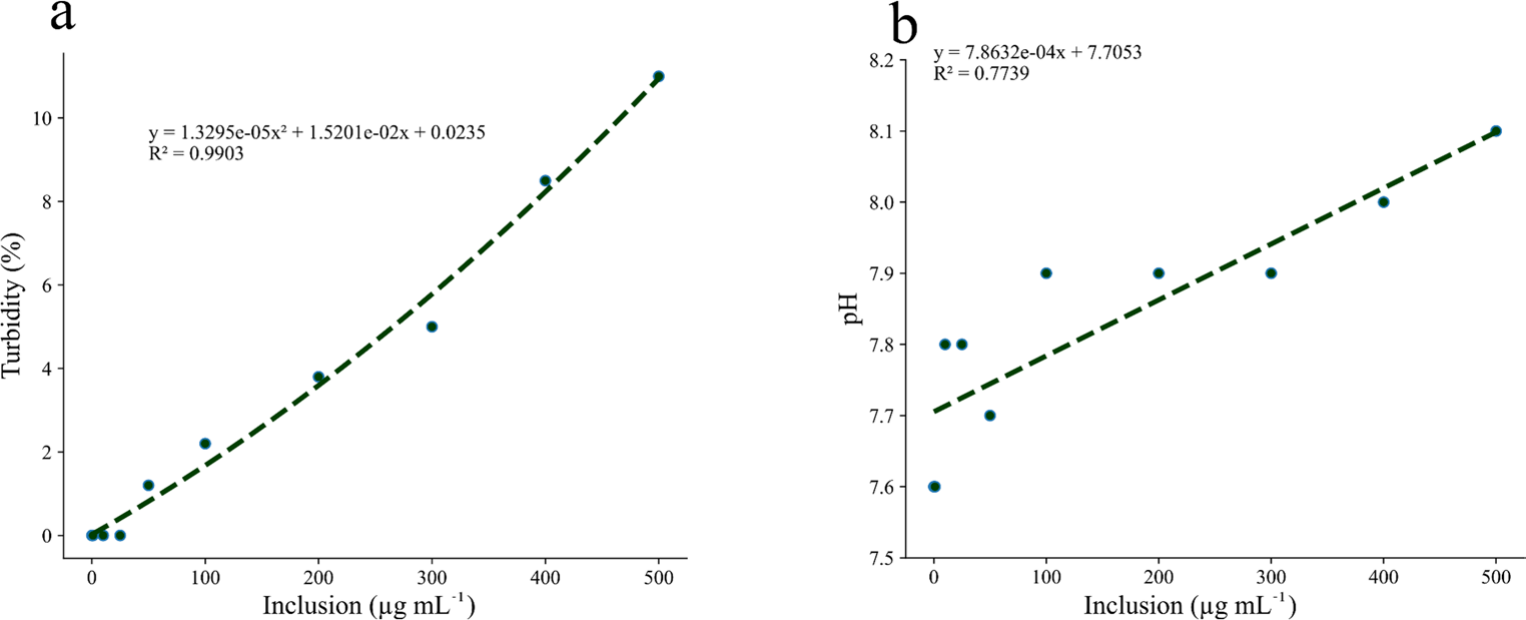
Turbidity (**a**) and pH (**b**) of water according to increasing doses of *C. guazumifolia* leaf decoction

Increasing doses of the decoction produced a dose-dependent rise in both turbidity and pH of the water (Fig. 2a-b).

**Fig. 3:**
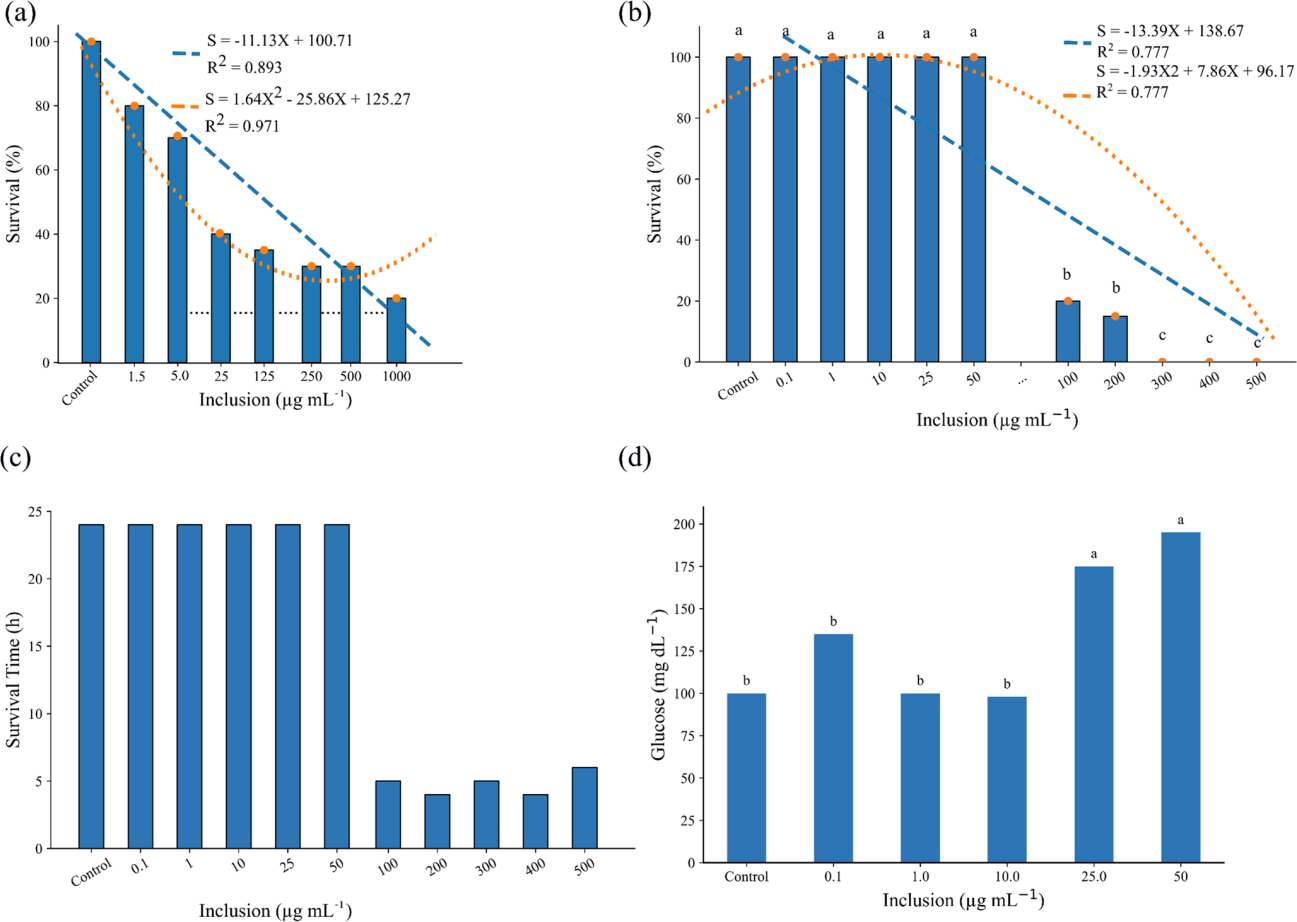
**a** Survival of *Artemia salina* exposed to increasing doses of *Campomanesia guazumifolia* leaf decoction. A significant reduction in survival was observed at concentrations above 25 µg mL⁻¹ compared with the control (Kruskal-Wallis: H = 21.1059; *p* = 0.0036). The lethal dose causing 50% mortality (LC_50_) was estimated at 23.29 µg mL⁻¹

Survival of *Artemia salina* was significantly affected by exposure to the decoction (Kruskal-Wallis: H = 21.1059; *p* = 0.0036). Survival decreased at concentrations ≥ 25 µg mL⁻¹, and the estimated LC_50_ was 23.29 µg mL⁻¹ (Fig. 3a).

In *G. pyrocephalus* juveniles, survival declined from 100 µg mL⁻¹ onward (Fig. 3b). Based on the mortality curve, the LC_50_ for fish was estimated at 50.03 µg mL⁻¹. Consistently, survival time decreased sharply at concentrations ≥ 100 µg mL⁻¹ (Fig. 3c). Plasma glucose increased in fish exposed to 25 µg mL⁻¹ or higher (Fig. 3d).

**Fig. 4:**
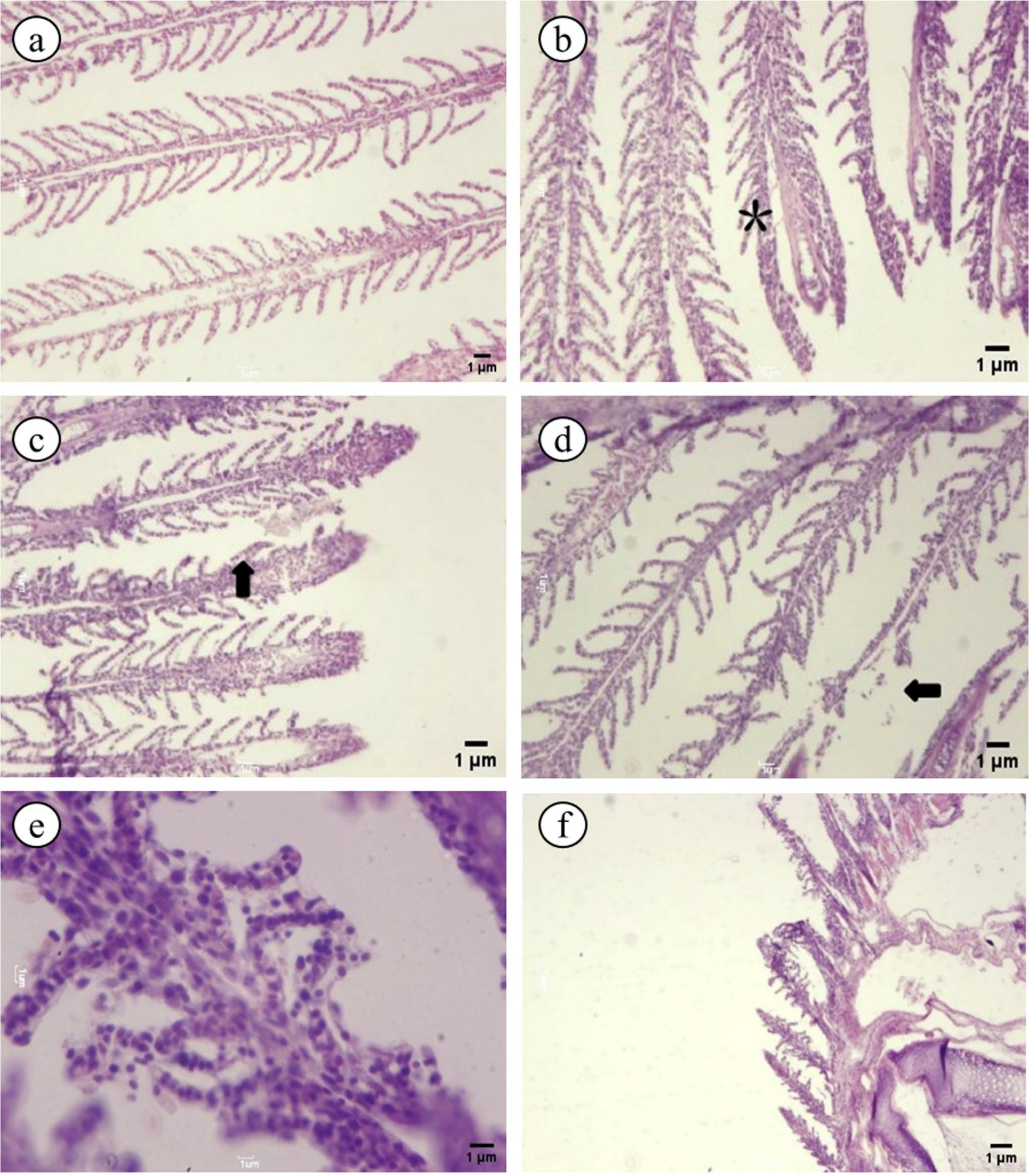
Photomicrographs of the gills of *Geophagus pyrocephalus* exposed to *C. guazumifolia* decoction for 24 h. (**a**) Control group: normal histological appearance of primary (PL) and secondary lamellae (SL). (HE; scale bar = 20 μm). (**b**) 0.1 µg mL⁻¹: progressive change characterized by mild hyperplasia of the secondary lamellar epithelium (*). (**c**) 1 µg mL⁻¹:regressive change showing epithelial detachment of the secondary lamellae (arrowhead). (**d**) 10 µg mL⁻¹: progressive change with interlamellar epithelial proliferation and apical fusion of secondary lamellae (arrowhead). (**e**) 25 µg mL⁻¹:regressive change showing necrosis of the basal matrix and extensive epithelial detachment. (HE; scale bar = 40 μm). (**f**) 50 µg mL⁻¹: severe regressive changes including lamellar disruption and complete epithelial lifting

The gills of control fish exhibited normal histological architecture (Ih = 0.8 ± 0.4), characterized by well-defined primary and secondary lamellae (Fig. 4a). Exposure to the *C. guazumifolia* decoction induced a dose-dependent increase in tissue alterations (*p* < 0.05; Table 2).

At lower concentrations (0.1 µg mL⁻¹), alterations were predominantly progressive changes, including mild epithelial hyperplasia and increased mucus production (Fig. 4b). As concentrations increased to 1.0 and 10 µg mL⁻¹, these progressive alterations intensified, leading to interlamellar epithelial proliferation and partial fusion of secondary lamellae (Fig. 4c-d), resulting in a moderate damage classification (Ih ranging from 12.5 to 18.4).

At the highest concentrations (25 and 50 µg mL⁻¹), severe regressive changes became evident, such as extensive epithelial detachment and necrosis of the supporting collagen matrix (Fig. 4e-f). These concentrations were associated with the highest histopathological indices (Ih = 32.6 ± 4.8 and 45.2 ± 6.5, respectively), classifying the tissue as severely altered. The circulatory disturbances, mainly congestion and hyperemia, were observed across all treatment groups but were most pronounced at higher exposure levels (Table 2).

**Table 2:** Histopathological reaction patterns and importance factors (w) used to calculate the Bernet Index (Ih​) for the gills of *G. pyrocephalus*

Treatment (µg mL⁻¹)	Total Index (Ih​)	Damage Classification
Control (0.0)	0.8 ± 0.4^a^	Unaltered tissue
0.1	4.2 ± 1.1^a^	Mildly altered
1.0	12.5 ± 2.3^b^	Moderately altered
10.0	18.4 ± 3.1^b^	Moderately altered
25.0	32.6 ± 4.8^c^	Severely altered
50.0	45.2 ± 6.5^d^	Severely altered

Values are expressed as mean ± standard deviation. Different letters in the same row indicate significant differences between treatments (*p* < 0.05) according to Dunn’s test

No significant differences were detected in growth performance or feed intake among treatments (Table 3). A treatment effect was observed for the condition factor (*p* = 0.050), with lower values recorded in fish fed diets containing the decoction compared to the control group. Hepatic alanine aminotransferase activity was significantly higher only in fish fed the diet containing 2000 mg kg⁻¹ of the decoction compared to the 250 mg kg⁻¹ and 500 mg kg⁻¹ treatments, while aspartate aminotransferase (AST) activity did not differ among treatments. In muscle tissue, SOD activity was significantly higher in fish fed 250 mg kg⁻¹ of the decoction compared with the other treatments (*p* < 0.05), whereas no significant differences were observed for CAT or liver SOD activity among treatments.

**Table 3:** Development and hepatic and antioxidant activity of *Geophagus pyrocephalus* fed diets containing *C. guazumifolia* decoction

Dietary inclusion of C. guazumifolia decoction (mg kg⁻¹)							
	Control	250	500	1000	2000	*P*	
**Performance**							
Weight gain (g)	0.352 ± 0.048	0.322 ± 0.083	0.310 ± 0.065	0.293 ± 0.050	0.280 ± 0.033	*0.266*	
Length gain (mm)	3.057 ± 0.151	3.043 ± 0.162	3.129 ± 0.138	3.129 ± 0.150	3.029 ± 0.076	*0.648*	
Condition factor	0.828 ± 0.056^a^	0.762 ± 0.102^b^	0.737 ± 0.084^b^	0.719 ± 0.071^b^	0.719 ± 0.031^b^	*0.634*	
**Liver activity** (IU mg^− 1^ protein)							
ALT	0.026 ± 0.013^ab^	0.025 ± 0.016^b^	0.025 ± 0.019^b^	0.032 ± 0.018^ab^	0.050 ± 0.009^a^	*0.027*	
AST	0.034 ± 0.012	0.035 ± 0.008	0.035 ± 0.010	0.034 ± 0.009	0.043 ± 0.008	*0.460*	
SOD	0.756 ± 0.399	1.241 ± 0.517	1.125 ± 0.696	0.830 ± 0.277	1.583 ± 0.943	*0.138*	
CAT	1.814 ± 0.711	2.561 ± 1.075	2.058 ± 1.001	1.595 ± 0.834	1.332 ± 0.312	*0.113*	
**Muscle Antioxidant Activity** (IU mg^− 1^ protein)							
SOD	1.093 ± 0.463^b^	2.713 ± 0.523^a^	1.200 ± 0.560^b^	1.047 ± 0.581^b^	1.298 ± 0.500^b^	*< 0.001*	
CAT	0.563 ± 0.253	0.592 ± 0.330	0.462 ± 0.257	0.327 ± 0.189	0.372 ± 0.094	*0.237*	

Different superscript letters indicate significant differences among treatments (Tukey’s test, *p* < 0.05). ALT: Alanine aminotransferase; AST: Aspartate aminotransferase; SOD: Superoxide dismutase in the liver; CAT: Catalase.

**Fig. 5:**
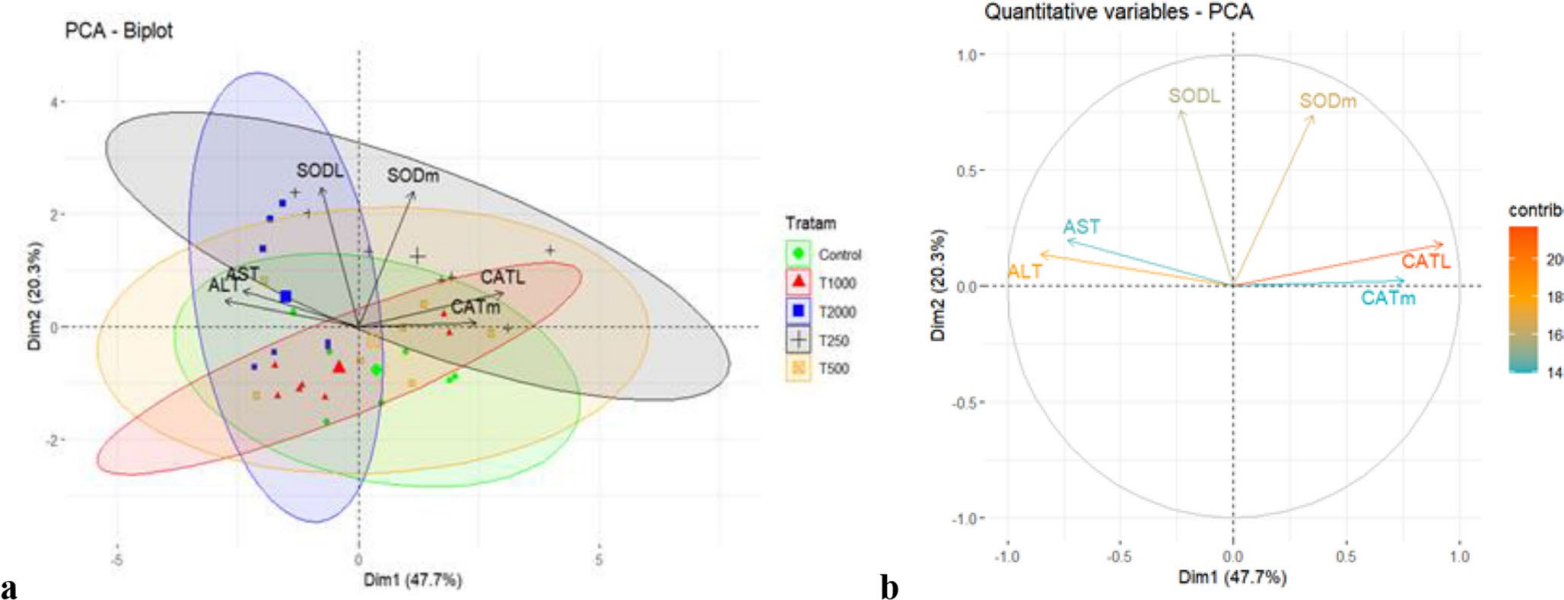
Principal component analysis (PCA) of biochemical and oxidative stress parameters in *Geophagus pyrocephalus* exposed to *C. guazumifolia* decoction. PC1 explained 47.7% of the total variance and PC2 explained 20.3%, together accounting for 68.0% of the total variability. (**a**) Biplot of Dimensions 1 and 2, where green dots represent the control group (diet without inclusion of *C. guazumifolia* leaf decoction), and black, orange, red, and blue dots represent the treatments with 250, 500, 1000, and 2000 mg kg⁻¹ of *C. guazumifolia* leaf decoction (T250, T500, T1000, and T2000), respectively. The rings correspond to the 95% confidence ellipses estimated using the treatment means. The explained variance for each dimension is shown in parentheses. (**b**) Correlation plot of quantitative variables and their contributions to each dimension. Abbreviations: CATm, catalase in muscle; CATL, catalase in liver; SODm, superoxide dismutase in muscle; SODL, superoxide dismutase in liver; ALT, alanine aminotransferase; AST, aspartate aminotransferase

Principal component analysis (PCA) effectively demonstrated a clear dose-response trajectory among the experimental groups (Fig. 5a-b). The first principal component (PC1) explained 47.7% of the total variance, revealing a strong positive correlation between the decoction concentration and specific physiological biomarkers. The second principal component (PC2) explained 20.3%, together accounting for 68.0% of the total variability.

Considering Fig. 5a and b jointly, liver catalase (CAT) activity, muscle superoxide dismutase (SOD), and alanine aminotransferase (ALT) were the variables contributing most strongly to the dimensional separation. A distinct shift from antioxidant protection to hepatic stress was observed: lower inclusion levels (250 mg kg⁻¹) were positively associated with increased muscle SOD activity and liver CAT activity, whereas higher concentrations (1000–2000 mg kg⁻¹) showed higher loadings for ALT and AST in the ordination space. These multivariate patterns are consistent with the univariate analysis, which confirmed significant increases in ALT for the T2000 group (*p* < 0.05) and elevated SOD activity for T250 (*p* < 0.05). The control, 500 mg kg⁻¹, and 1000 mg kg⁻¹ groups exhibited intermediate and similar multivariate profiles, reinforcing the identification of 250 mg kg⁻¹ as a beneficial threshold and 2000 mg kg⁻¹ as a concentration inducing metabolic stress.

## Discussion

Flavonoids are widely reported in *Campomanesia* species (Schmeda-Hirschmann 1995; Arruda 2013; Ferreira et al. 2013; Michel et al. 2013; Catelan et al. 2018; Castro et al. 2023). Consistent with these findings, five glycosylated flavonoids were identified in the decoction of *Campomanesia guazumifolia* leaves using MS and MSn fragmentation patterns: quercetin pentoside, quercetin deoxyhexoside, myricetin deoxyhexoside, myricetin hexoside, and myricetin deoxyhexoside-gallate (Table 1). Glycosylated flavonoids represent a major class of plant phenolics, often occurring as sugar-conjugated metabolites (Simões et al. 2017). Similar flavonoid profiles have been reported for other *Campomanesia* species (Arruda 2013; Lescano et al. 2023), supporting the chemical pattern observed in the present decoction.

Although phenolic compounds are widely recognized for their antioxidant properties, flavonoids may also exhibit pro-oxidant behavior depending on concentration and environmental conditions. At higher concentrations, these compounds can promote the formation of reactive oxygen species, leading to oxidative imbalance rather than protection (Jomová et al. 2017; Jomova et al. 2019; Simunkova et al. 2021). This pattern suggests a dose-dependent dual effect (hormetic response), in which low concentrations may induce adaptive antioxidant responses, whereas higher concentrations result in oxidative stress and toxicity.

Toxicity assessment is an essential step in validating the safety of natural extracts for therapeutic or nutraceutical applications (Arruda 2013; Viscardi et al. 2017; Silva et al. 2016; Castro and Cardoso 2023). While some *Campomanesia* species have been shown to be safe in vivo systems such as *C. xanthocarpa* (Silva et al. 2016) d *adamantium* (Viscardi et al. 2017) the present study demonstrated pronounced toxicity of *C. guazumifolia* leaf decoction for both *Artemia salina* (LD₅₀ = 23.29 µg mL⁻¹) and *Geophagus pyrocephalus* juveniles (LD₅₀ = 50.03 µg mL⁻¹). According to the classification proposed by Sandoval et al. (2020), these values indicate high toxicity (LD₅₀ < 100 µg mL⁻¹). Although flavonoid derivatives such as quercetin and myricetin were identified, it is not possible to attribute the observed toxic effects exclusively to these compounds. The decoction may contain additional metabolites not detected in the present analysis, which could also contribute to the biological responses observed.

This result contrasts with reports of low or absent toxicity for *C. guazumifolia* aqueous infusions in *Artemia* (LD₅₀ > 900 µg mL⁻¹) (Arruda 2013; Castro et al. 2023). Such discrepancy likely arises from differences in extraction procedures, as decoction due to prolonged heating may extract a broader or more concentrated set of compounds. Additionally, differences in extract concentration may have increased the exposure to bioactive compounds, potentially exceeding physiological tolerance limits. This interpretation is consistent with Silva (2011), toxicity is likely associated with the chemical profile of the decoction.

Despite the marked toxicity observed here, other *Campomanesia* extracts have shown biological safety at moderate doses. For example, Guerrero et al. (2010) reported no renal or hepatic toxicity in rats exposed to hydroethanolic extract of *C. pubescens* at 250–500 mg kg⁻¹, suggesting that toxicity depends strongly on solvent, extraction temperature, and plant organ used. Therefore, the high toxicity of the decoction observed in the present study likely reflects the specific chemical profile yielded by this preparation method rather than an inherent toxic nature of the species.

Gill tissue is in direct contact with the aquatic environment and is highly sensitive to chemical stressors. The gill lesions observed in *G. pyrocephalus* are consistent with responses to pollutants and xenobiotics reported in the literature (Garcia-Santos et al. 2007; Cantanhêde et al. 2014). This sensitivity was quantitatively reflected in the significant increase of the histopathological index (Ih​), which shifted from a status of “unaltered” in the control to “severely altered” at higher concentrations, according to the Bernet et al. (1999). Such morphological changes, including mucus hypersecretion and reduced respiratory surface area, can compromise gas-exchange efficiency (Karlsson-Norrgren et al. 1985; McDonald and Wood 1993). These alterations can reduce oxygen uptake efficiency, increase energetic costs associated with ventilation, and impair ionic and acid-base balance, ultimately compromising fish survival and performance. The present findings therefore indicate that *C. guazumifolia* leaf decoction acts as a gill stressor at the tested inclusion levels, inducing structural damage at sublethal concentrations as evidenced by the high Ih​ values recorded prior to mortality.

Regarding genotoxic endpoints, micronucleus formation is a well-established indicator of clastogenic or aneugenic exposure in fish (Udroiu 2006; Dalzochio et al. 2016; Braga et al. 2021; Tamanho et al. 2022). Genotoxic and histopathological responses do not always occur simultaneously (Dalzochio et al. 2016), suggesting that different toxicity pathways may be activated depending on the compound class and exposure level. Some flavonoids can exhibit pro-oxidant or DNA-reactive effects at higher concentrations, which could theoretically contribute to genotoxicity, however, this hypothesis would require targeted confirmation in future studies.

The biological effects of plant extracts depend heavily on phytochemical composition, which varies with environmental conditions, processing, and extraction technique (Arruda 2013; Carneiro et al. 2023; Castro et al. 2023). In aquaculture, plant-derived bioactives have attracted increasing attention for their potential to modulate metabolic and oxidative processes (Honorato et al. 2021). Positive physiological effects have been documented in several species, including improvements in growth performance in Nile tilapia supplemented with *Campomanesia* fruit extract (Moraes et al. 2025) and enhanced longevity in ornamental fish receiving nutraceutical supplementation (Reges et al. 2024).

The altered activities of AST and ALT observed here reflect hepatic responses to the decoction, as increases in these enzymes indicate cellular damage or metabolic disturbance (Honorato et al. 2017). Additionally, elevations in antioxidant enzymes (CAT and SOD) were detected, consistent with reports of enhanced oxidative metabolism in fish exposed to phenolic-rich diets (França et al. 2022; Cruz et al. 2023; Siqueira et al. 2024). Viscardi et al. (2017) highlighted the strong free-radical-scavenging properties of flavonoids in *C. adamantium*, which may underlie the oxidative adjustments observed. The stability of flavonoids in the supplemented diets and the PCA results showing clear separation of treatments driven largely by oxidative biomarkers reinforce the role of these compounds in modulating antioxidant pathways in *G. pyrocephalus*.

Furthermore, the biological relevance of these metabolic shifts is supported by the semi-quantitative histopathological analysis. The integration of semi-quantitative histopathological analysis with multivariate data further elucidates the biological impact of *C. guazumifolia* decoction. The calculation of the histopathological index allowed for a nuanced interpretation of tissue integrity that aligns with the PCA results. While the PCA indicated a shift toward metabolic stress at the highest inclusion level (2000 mg kg⁻¹), the histopathological findings provided the morphological evidence of this transition. Specifically, the increase in I h in the liver of the T2000 group, characterized by severe epithelial disruption, correlates with the higher loadings of ALT and AST observed in the ordination space. This convergence of data suggests that the enzymatic leakage into the bloodstream is a direct consequence of the structural alterations. Conversely, the low Ih values and the prevalence of unaltered reaction patterns in the T250 group reinforce the safety and the antioxidant-promoting role of lower inclusion levels, as evidenced by the SOD and CAT vectors in the PCA biplot.

Overall, the findings demonstrate that although *C. guazumifolia* leaf decoction contains flavonoids with recognized biological potential, its toxicity at relatively low concentrations warrants caution. The physiological, histological, and biochemical alterations observed highlight the importance of selecting appropriate extraction methods and inclusion levels when considering plant-derived bioactives for aquaculture applications.

## Conclusion

This study provides evidence that the *C. guazumifolia* leaf decoction modulates antioxidant responses, particularly at lower dietary inclusion levels. However, the observed increases in CAT and SOD activity may also reflect compensatory responses to oxidative stress rather than direct protective effects. Escalating doses elicited clear toxicological responses, including hepatic dysfunction, pronounced histopathological alterations, and reduced survival rates, underscoring a narrow margin between beneficial and deleterious effects. Higher doses elicited clear toxicological responses, including hepatic dysfunction, pronounced histopathological alterations, and reduced survival rates, indicating a narrow margin between potential benefits and adverse effects. Among the evaluated concentrations, the inclusion of 250 mg kg⁻¹ can be considered safe under the tested conditions, although further studies addressing long-term exposure and chronic effects are required.

## Data Availability

The datasets generated and analyzed during this study are not publicly available, but can be obtained from the corresponding author upon reasonable request.
